# Leber’s hereditary optic neuropathy - Case report

**Published:** 2018

**Authors:** Raluca Eugenia Iorga, Ruxandra Mihailovici, Manuela Ramona Ozturk, Dănuţ Costin

**Affiliations:** * Department of Ophthalmology, ”N. Oblu” Clinical Emergency Hospital, Iaşi, Romania; ** Department of Ophthalmology, ”Gr. T. Popa” University of Medicine and Pharmacy, Iaşi, Romania

**Keywords:** Leber’s hereditary optic neuropathy, mitochondrial DNA test, optic coherence tomography, idebenone

## Abstract

Leber’s hereditary optic neuropathy is the most common mitochondrial condition and is characterized by bilateral, painless, subacute visual loss that develops during young adult life. LHON is a rare condition and this lack of knowledge can make doctors suspect and treat for other causes of vision loss. Typically, a series of tests are performed to confirm LHON diagnosis or exclude any other conditions. We presented the case of two brothers, HB, of 40 years old and HF, of 38 years old, who presented with a decrease in visual acuity in both eyes. The patients had been diagnosed with optic atrophy of unknown cause a long time ago, but no further investigations were made. They were treated with corticosteroids, antioxidants and vasodilators, but with no significant benefit. A blood test of the mitochondrial DNA, a magnetic resonance imaging and an optic coherence tomography of the optic nerve and macula were part of the following assessment of our patients. The mitochondrial DNA analyses revealed the 3460 G>A mutation on the mtND1 gene in both patients. Based on the medical history, the fundus aspect, the optic coherence tomography and the paraclinical investigations of the diagnosis of Leber’s hereditary optic neuropathy were established in both patients. We started the treatment with idebenone and we evaluated the patients after three months.

Abbreviations: LHON = Leber’s hereditary optic neuropathy, mtDNA = mitochondrial DNA, VA = visual acuity, CF = count fingers, OCT = optical coherence tomography, RNFL = retinal nerve fiber layer, GCL = ganglion cells layer, MS = multiple sclerosis, MRI = magnetic resonance imaging, MTI = magnetization transfer imaging, MTR = magnetization transfer ratio

## Introduction

Leber’s hereditary optic neuropathy (LHON) was first described in 1871 by the German ophthalmologist Theodore Leber and was subsequently named after him. LHON is characterized by bilateral, painless, subacute visual loss that develops during young adult life. 

LHON is the most common mitochondrial condition and about 45 mutations have been linked to LHON. A person may carry a mitochondrial DNA (mtDNA) mutation without experiencing any signs or symptoms of vision loss; therefore, it is hard to predict which members of a family who carry a mutation will eventually become affected [**1**].

Clinically, most patients with LHON go through pre-symptomatic ophthalmoscopic changes before the acute phase, characterized by visual loss. LHON mainly affects the central vision, leading to large bilateral centrocecal scotoma. By 6 months after onset, optic atrophy is evident and visual loss stabilizes. The chronic phase is reached by 1 year after onset. In some rare cases, patients do experience a significant recovery of vision and this is more likely to happen if you have the 14484 mutation [**2**].

If LHON is suspected, a blood test can determine if an individual has one of the primary mutations. However, LHON is a rare condition and most doctors can easily pass over this mtDNA test. This lack of knowledge can make doctors suspect and treat for other causes of vision loss. Typically, a series of tests are performed to confirm LHON diagnosis or exclude any other conditions.

## Case report

We present the case of two brothers, HB, of 40 years old and HF, 38 years old, who presented with a decrease in visual acuity. From the medical history, we found that the onset of symptoms was about 3 years before in HB and 5 years before in HF, when a diagnosis of optic atrophy of unknown cause was made. They followed treatment with corticosteroid intravenously iv in the beginning and then vasodilators at every 3 months, and antioxidants but with no significant benefit.

**Fig. 1:**
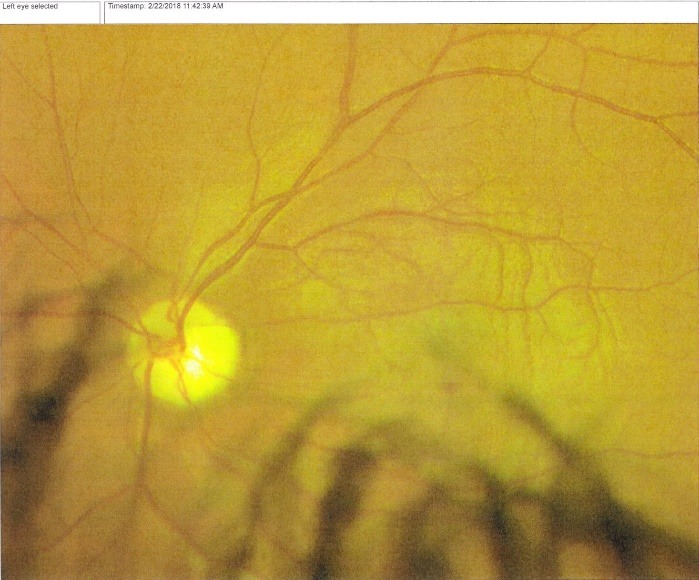
Fundus left eye: optic atrophy, narrow vessels, no macular reflex

**Fig. 2:**
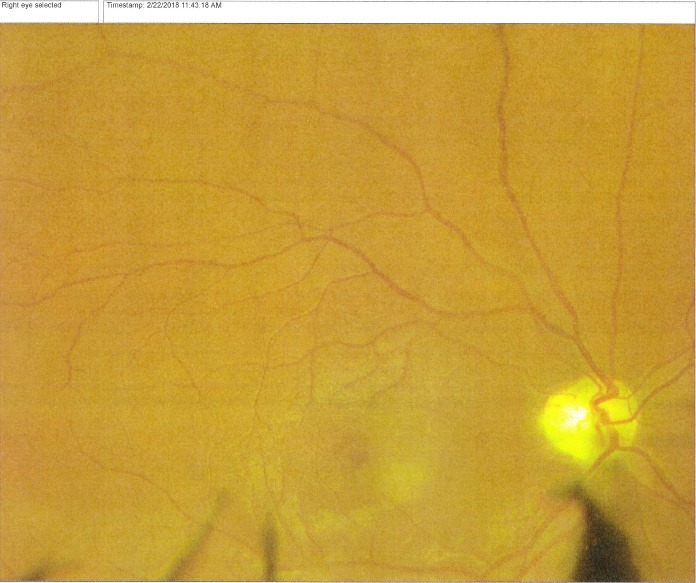
Fundus right eye: optic atrophy, narrow vessels, no macular reflex

At presentation, the patient HF had best corrected visual acuity (VA) in both eyes count fingers (CF) at 2 m, normal intraocular pressure IOP (15 mmHg). On slit lamp examination, the findings of the anterior pole were within normal limits. The fundus of each eye was examined after mydriasis with tropicamide and revealed optic atrophy in both eyes, with narrow vessels and no macular reflex (**Fig. 1**,**2**). The patient HB presented with a VA of CF at 1m and an IOP within normal limits. The anterior segment examination at the slit lamp was normal. Fundus examination showed a bilateral optic atrophy, narrowed vessels and no macular reflex, similar with the fundus examination of his brother. In this context, we thought of the multiple causes of optic atrophy, but taking into account the young age, the hereditary aspect and no other medical history and risk factors, we made a stage diagnosis of Hereditary optic neuropathy.

The usual blood test and inflammatory tests were within normal limit. A blood test of the mtDNA, a magnetic resonance imaging MRI and an optic coherence tomography of the optic nerve and macula were part of the following assessment of our patients. The mtDNA analyses revealed the 3460 G>A mutation on the mtND1 gene in both patients.

**Fig. 3:**
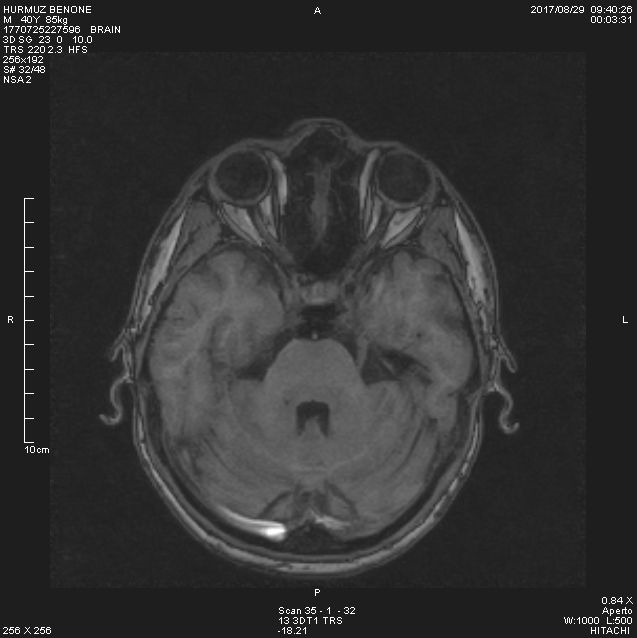
MRI HB - The MRI in our patient showed that the mean values of optic nerve volumes were significantly lower

The MRI exam in HB showed (**Fig. 3**):

- in the sequence axial 3 D, the optic nerves had similar thickness, but slightly reduced in 3 segments (retrobulbar 2 mm - normal 1.5-4.4 mm, the middle segment of the intraorbital part 1.5 mm - normal 1.8-2.5 mm, in the optic channel 2 mm - normal 3.6-6.5 mm, prechiasmatic 4 mm - normal 4.7-5.5 mm)

- the symmetric enlargement of the intergiral grooves, mostly parieto-occipital

- no pathologic contrast fixation. 

**Fig. 4:**
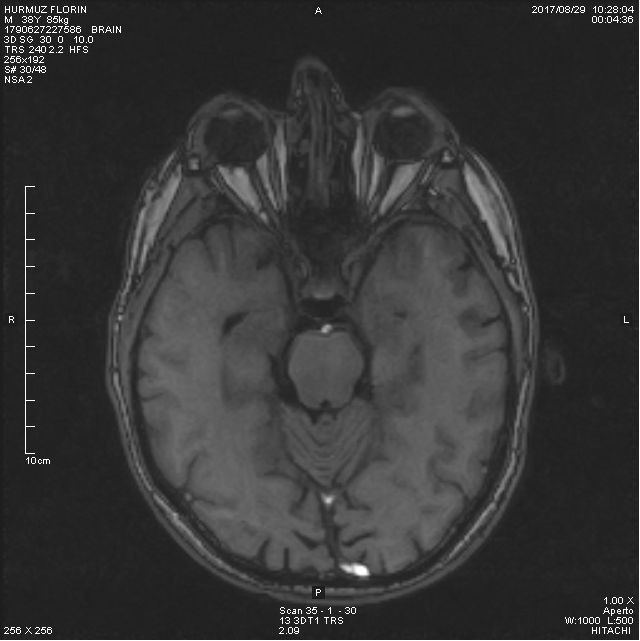
MRI in HF - The MRI in our patient showed that the mean values of optic nerve volumes were significantly lower

The MRI in HF showed (**Fig. 4**):

- in the sequence axial 3 D, the optic nerves had similar thickness, but slightly reduced in only 2 segments (retrobulbar 2 mm - normal 1.5-4.4 mm, the middle segment of the intraorbital part 2 mm

- normal 1.8-2.5 mm, in the optic channel 3.3 mm - normal 3.6-6.5 mm, prechiasmatic 4 mm - normal 4.7-5.5 mm)

- the symmetric enlargement of the intergiral grooves

- no pathologic contrast fixation

- asymmetric enlargement of the perivascular spaces with milimetric cysts. 

**Fig. 5:**
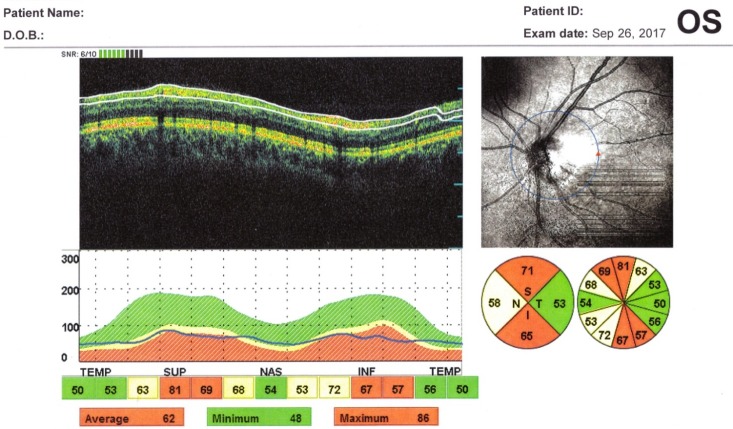
OCT left eye: reduced peripapillary RNFL in the superior, inferior, and nasal segments

**Fig. 6:**
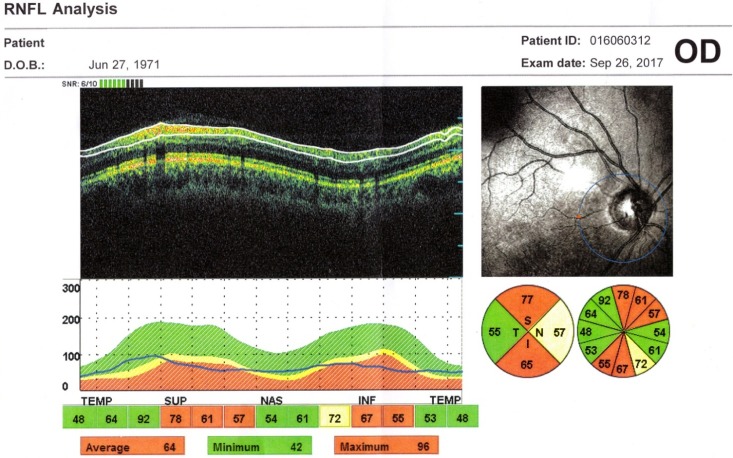
OCT right eye: reduced peripapillary RNFL in the superior, inferior, and nasal segments

**Fig. 7:**
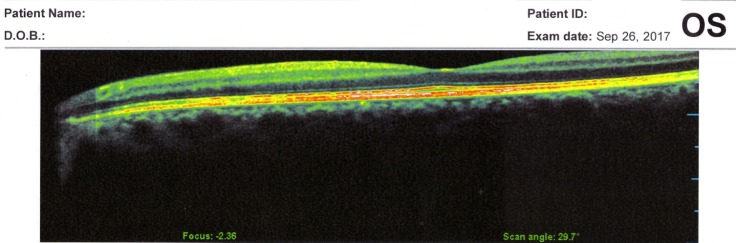
OCT macula normal

**Fig. 8:**
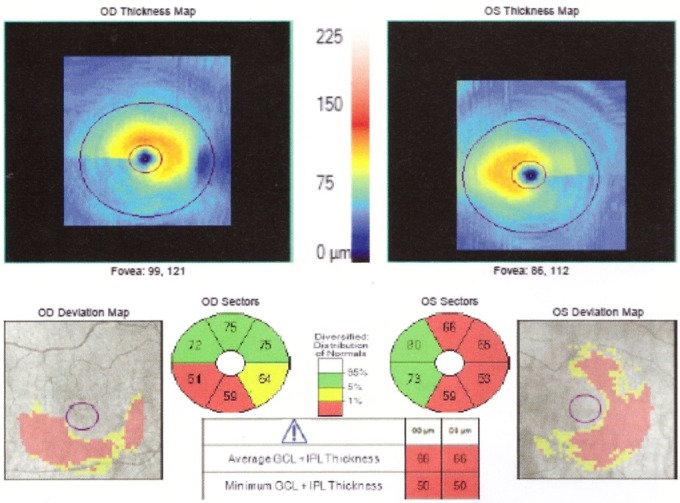
OCT macula affected GCL in both eyes

The OCT exam revealed a normal size optic disc, but with severely affected retinal nerve fiber layer RNFL in both eyes of the two patients, mostly in the superior, inferior, and nasal quadrants (**Fig. 5**,**6**). The macular region was normal, without microcystic degeneration (**Fig. 7**), but with affected ganglion cells layer (**Fig. 8**).

Based on the medical history, the fundus aspect, the OCT and the paraclinical investigations (mtDNA, MRI, blood tests), the diagnosis of Leber’s hereditary optic neuropathy were established in both patients.

We started a treatment with systemic idebenone, 6 tablets a day (900 mg/ day), for 1 year. We examined the patients 3 months later. Subjectively, the 2 patients were pleased with an improvement in clarity and contrast sensitivity. In HF, we noticed a VA of CF at 4 m, with no modification on fundus examination and OCT. In HB, the VA recovery was slightly smaller, only CF in 2 m, with the same fundus and OCT. The usual blood test in both patients was within normal limits.

## Discussions

Hereditary optic neuropathies are diseases affecting the optic nerve. They may appear as isolated optic neuropathies or in association with systemic diseases. LHON is an inherited form of vision loss, which leads to the selective loss of retinal ganglion cells and axons, in particular of the papillomacular bundle [**3**]. Although this condition usually begins in a person’s teens or twenties, rare cases may appear in early childhood or later in adulthood. 

LHON is a maternally inherited disorder that has been associated with mtDNA mutations. The most common is the 11778 mutation, accounting for about 50% of all LHON cases. About 45% of the remaining LHON cases are 14484 or 3460 mutations [**4**]. Changes in mitochondrial DNA mtDNA appear in every generation of a family and can affect both males and females, but fathers do not pass mitochondrial traits to their children. The mtDNA mutation is necessary but not sufficient to induce the disease. Other, still poorly defined, genetic, or environmental factors may be implicated. About 40% of the individuals affected with LHON do not have a clear family history of this condition [**5**].

In the pre-symptomatic stage, the ophthalmoscopic changes are peripapillary microangiopathy, small vessel tortuosity, swelling of the retinal nerve fiber layer RNFL. A sudden painless blurring and clouding of vision are usually the first symptoms of the acute phase of LHON. These vision problems may begin in one eye or simultaneously in both eyes. If vision loss starts in one eye, the other eye is usually affected within several weeks or months. The rate of progression can vary from rapid to over 2 years but most people are severely impaired by 3 or 4 months. Over time, vision in both eyes worsens with a severe loss of visual acuity and color vision, due to optic atrophy [**2**].

OCT has been used to investigate the RNFL of LHON unaffected carriers, as well as LHON affected patients in all stages. In healthy carriers, the OCT showed RNFL thickening in temporal quadrants and in asymptomatic men in the inferior sector [**6**]. RNFL thickening is correlated with axonal edema and mitochondrial redistribution at dysfunctional ganglion cells, affecting the prelaminar unmyelinated portion of the optic nerve axons [**7**]. In the acute phase and in the first 6 months of onset, the RNFL analysis shows thickening in the upper and lower segments, compared to the control group [**8**,**9**]. At the atrophic stage, a RNFL thinning appears in all segments. The optic disc analysis showed that carriers had larger discs than the affected patients. The smaller optic nerve head in the affected patients may be related to the mechanical compression of the papillomacular bundle fibers, which represent the trigger dysfunction and axonal death [**10**]. The OCT analysis of macular thickness showed a thinning of the GCL at the early stage in the sectors of the inner ring [**11**]. 

In our patients, the OCT exam revealed normal size optic disc, but with severely affected RNFL in both eyes mostly in the superior, inferior, and nasal quadrants and with affected GCL.

The severity of optic nerve pathology in LHON is measurable in vivo using MRI and magnetization transfer imaging MTI. A subtype of LHON presents additional clinical and MRI aspects indistinguishable from those of multiple sclerosis (MS) (LHON-MS). In patients with LHON or LHON-MS, an assessment was made of the severity of optic nerve damage, using MRI and MT, and the presence and extent of macroscopic and microscopic pathology in the brain and cervical cord, using MRI and MT ratio (MTR). The mean values of optic nerve volumes and MTR were significantly lower in patients with LHON than in healthy controls [**12**]. An association between LHON and MS has been suggested and evidence for such an association has been found by recent studies with MRI [**13**]. In another study, 26% of the patients with LHON had white matter lesions. Additionally, a quantitative MRI study of patients with LHON found abnormalities of the normal-appearing brain tissue consistent with that previously reported in MS. A hypothesis as to why LHON may be a risk factor for MS is that mitochondrial defects trigger the autoimmune process. A further possibility is that mitochondrial dysfunction is a final common pathway in neural damage [**14**]. 

The MRI in our patients showed that the mean values of optic nerve volumes were significantly lower than in healthy controls.

Regarding the treatment, some patients are self-medicating with a range of mostly antioxidant food supplements. Early studies testing a range of antioxidants and other supplements including curcumin, lutein, brimonidine, and others have proved inconclusive and most scientific research into this area seems to have ceased. Idebenone (Raxone(®)), a short-chain benzoquinone, has been the only disease-specific drug approved to treat visual impairment in adolescents and adults with LHON, since September 2015. The mechanism of action of idebenone involves its antioxidant properties and ability to act as a mitochondrial electron carrier. Idebenone overcomes mitochondrial complex I respiratory chain deficiency in patients with LHON by transferring electrons directly to mitochondrial complex III (by-passing complex I), thereby restoring cellular energy production and re-activating inactive, but viable retinal ganglion cells, which ultimately prevent further vision loss and promote vision recovery. 

Klopstock T conducted a 24-week multi-centre double blind, randomized, placebo-controlled trial in 85 patients with LHON. This first randomized controlled trial in the mitochondrial disorder provides evidence that patients with discordant visual acuities are the most likely to benefit from idebenone treatment, which is safe and well tolerated. They investigated the treatment effect among patients with the 11778G>A and 3460G>A mutations [**15**]. 

Heitz et al. described that in mice, idebenone penetrated into the eye at concentrations equivalent to those that protected retinal ganglion cells from complex I dysfunction in vitro. Consequently, they investigated the protective effect of idebenone in a mouse model of LHON, whereby mitochondrial complex I dysfunction was caused by exposure to rotenone. In this model, idebenone protected against the loss of retinal ganglion cells, reduction in retinal thickness and gliosis. Furthermore, consistent with this protection of retinal integrity, idebenone restored the functional loss of vision in this disease model [**16**]. After a 3 months treatment, we observed a slightly increase of VA and an improvement in contrast sensitivity in our patients. 

**Case particularity**

The patients were diagnosed a long time ago with optic atrophy of unknown cause, but no further investigations were made. They were treated with corticosteroids, antioxidants and vasodilators, but with no significant benefit. A blood test, the mtDNA test, helped us make the correct diagnosis and the new treatment with idebenone gave the patients hope for visual improvement.
